# Nanocavity-induced trion emission from atomically thin WSe_2_

**DOI:** 10.1038/s41598-022-20226-3

**Published:** 2022-09-23

**Authors:** Zhuo Wang, Yuanda Liu, Dao Chen, Zixuan Wang, Mohamed Asbahi, Soroosh Daqiqeh Rezaei, Jie Deng, Jinghua Teng, Andrew T. S. Wee, Wenjing Zhang, Joel K. W. Yang, Zhaogang Dong

**Affiliations:** 1 International Collaborative Laboratory of 2D Materials for Optoelectronics Science and Technology of Ministry of Education, Institute of Microscale Optoelectronics, Shenzhen University, Shenzhen, 518060 China; 2 Institute of Materials Research and Engineering, A*STAR (Agency for Science, Technology and Research), 2 Fusionopolis Way, #08-03 Innovis, Singapore, 138634 Singapore; 3 Department of Electrical Engineering, The Pennsylvania State University, University Park, PA 16802 USA; 4 Materials Research Institute, The Pennsylvania State University, University Park, PA 16802 USA; 5 Department of Physics, National University of Singapore, 2 Science Drive 3, Singapore, 117551 Singapore; 6 Singapore University of Technology and Design, 8 Somapah Road, Singapore, 487372 Singapore; 7 Department of Materials Science and Engineering, National University of Singapore, 9 Engineering Drive 1, Singapore, 117575 Singapore

**Keywords:** Materials science, Optics and photonics, Physics

## Abstract

Exciton is a bosonic quasiparticle consisting of a pair of electron and hole, with promising potentials for optoelectronic device applications, such as exciton transistors, photodetectors and light emitting devices. However, the charge-neutral nature of excitons renders them challenging to manipulate using electronics. Here we present the generation of trions, a form of charged excitons, together with enhanced exciton resonance in monolayer WSe_2_. The excitation of the trion quasiparticles is achieved by the hot carrier transport from the integrated gold plasmonic nanocavity, formed by embedding monolayer WSe_2_ between gold nanoparticles and a gold film. The nanocavity-induced negatively charged trions provide a promising route for the manipulation of excitons, essential for the construction of all-exciton information processing circuits.

## Introduction

Two-dimensional (2D) transition metal dichalcogenides (TMDCs) are atomically thin sheets that exhibit unique optical and electronic properties^1–4^. Unlike conventional semiconductors and few-layer TMDCs, electrons and holes in monolayer TMDCs are tightly bound at the energy degenerate ± K valleys^5,6^. These strong Coulombic interactions and reduced dielectric screening effect give rise to free excitons and charged excitons (trions)^7–9^. Due to the high (> 100 meV) exciton binding energy^10^, the free excitons remain stable and display strong exciton resonance even at room temperature, and thus determine the optical response and light emission properties of TMDCs. However, the trion is not easily observed as its binding energy is much smaller than the free excitons^11,12^.

Electrostatic tuning^13^, chemical doping^14^, and cryogenic cooling^15^ have been used to observe trion-related photon emission. Distinct from charge-neutral excitons with in-plane dipole orientation and short light emission lifetime (*i.e.,* several picoseconds), trions have been recently reported to have a partially out-of-plane dipole orientation^16^, longer light emission lifetime (tens of picoseconds) and larger valley polarization^17^. These unique characteristics of trions have great potential in implementing on-chip optical communications and valleytronic devices^16,18^.

To enhance photoemission from trions, some preliminary investigations based on dielectric microresonators^16^ and plasmonic nanocavities^19,20^ have been reported by exploring the enhancements of light absorption and emission in TMDCs via enhanced light-matter interaction. As plasmonic field enhancement is highest when the gap between metallic nanoantennas is decreased to the nanometer scale^21,22^, it is enticing to investigate the mechanism of trion emission from atomically thin WSe_2_ sandwiched in plasmonic cavity resonators with nanometer gaps, where such a system has not been thoroughly investigated so far.

In this work, we present detailed investigation of trion formation and significantly enhanced exciton resonance with energy-level splitting in monolayer WSe_2._ Plasmonic nanocavity with a few nm gap was fabricated by spreading a monolayer of gold nanoparticles (AuNPs) with 2-nm ligands as outer shells onto monolayer WSe_2_ flakes placed on an Au-coated substrate. *Z*-polarized localized gap plasmons were excited inside the nanogaps between AuNPs and the Au film. The strongly localized gap plasmons enable the generation of hot carriers on plasmonic nanostructures, which transport to WSe_2_ via quantum tunneling through the ligands coated on the AuNPs. The plasmonic nanocavity induced negatively charged trions are expected to provide a candidate route for manipulating the excitons, which is essential for the construction of all-exciton information processing circuits.

## Results and discussion

**Figure 1:**
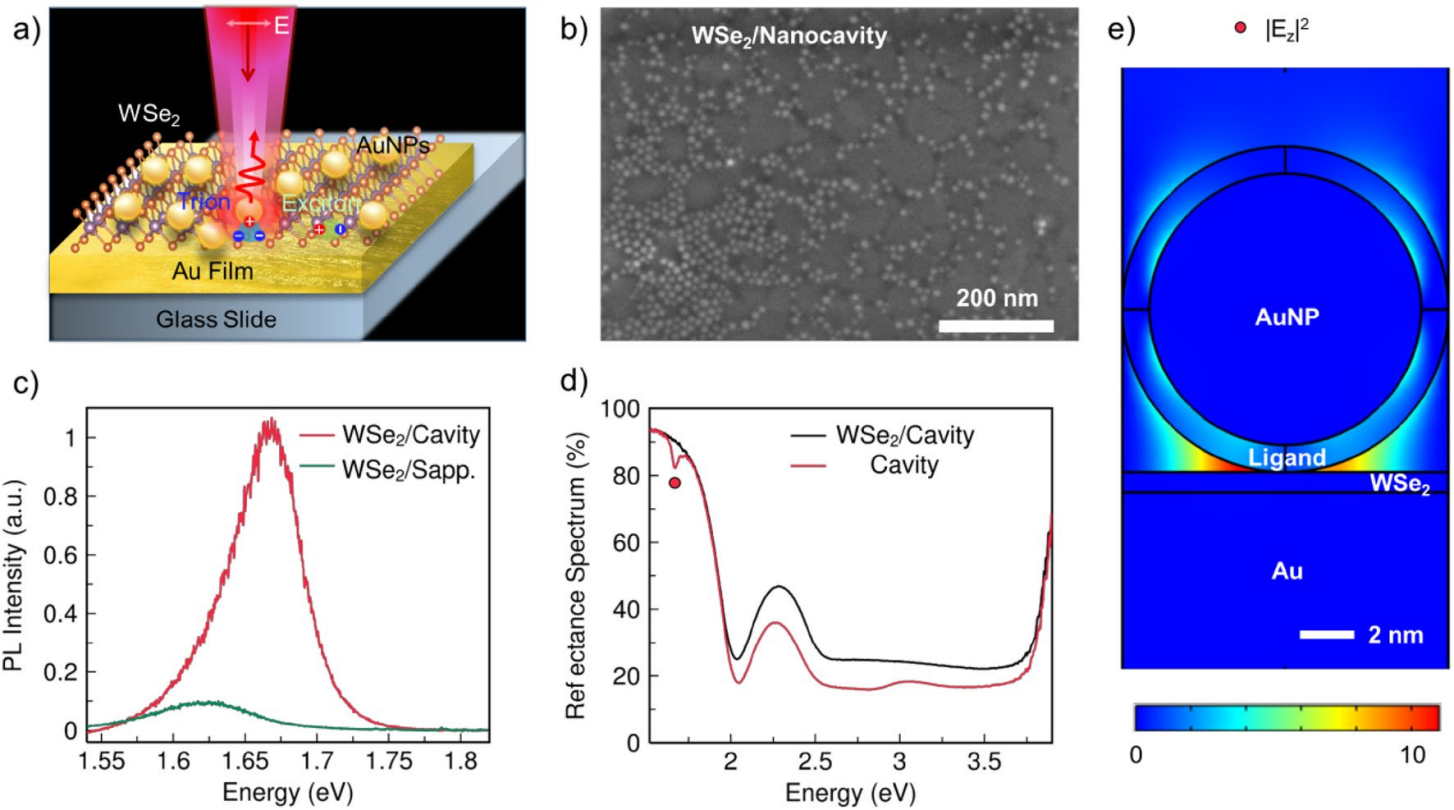
Monolayer WSe_2_ embedded in a plasmonic nanocavity and characterizations. (**a**) Schematic of monolayer WSe_2_ embedded in a plasmonic nanocavity comprising gold nanoparticles (AuNPs) and gold-film substrate, with enhanced PL emission being observed. (**b**) Representative scanning electron microscope (SEM) image of monolayer WSe_2_ embedded in nanocavity (WSe_2_/Nanocavity). (**c**) PL spectra of monolayer WSe_2_ in nanocavity and monolayer WSe_2_ on sapphire with an emission peak at 1.67 eV and 1.62 eV, respectively. (**d**) Reflectance spectra of nanocavity with and without monolayer WSe_2_. The red dot at 1.68 eV denotes the energy of localized plasmon resonance. (**e**) Finite-difference time-domain (FDTD) simulation of the enhanced electric displacement intensity, |*E*_*z*_|^2^, inside the monolayer WSe_2_ due to the localized vertical gap plasmons.

Enhanced exciton resonance and photoluminescence (PL) emission in WSe_2_ was demonstrated using the plasmonic nanocavity. The monolayer WSe_2_ was embedded inside the gap of the plasmonic nanocavity as shown in Fig. 1a. With the nanoparticles on mirror geometry configuration, the embedded monolayer WSe_2_ can achieve strong exciton resonance and PL emission. The monolayer WSe_2_ flakes were grown on a sapphire substrate by chemical vapor deposition (CVD) and immediately transferred onto an ultrasmooth template-stripped gold substrate using a wet-transfer technique^22,23^. The ~ 10 nm diameter AuNPs were deposited onto the gold substrate via a self-assembly method (details in the “Methods” section)^24^. The quality and layer number of WSe_2_ were determined using optical microscopy, Raman (Fig. S1) and PL spectroscopy before the transferring process.

The scanning electron microscopy (SEM) image of the hybrid structure is shown in Fig. 1b. The AuNPs have a spherical morphology with a uniform diameter of ~ 10 nm, where these AuNPs are tightly anchored on the surface of WSe_2_, especially in areas with defects and cracks. PL spectra at different places from WSe_2_ embedded on nanocavity (WSe_2_/Nanocavity) and WSe_2_ grown on sapphire (WSe_2_/Sapphire) were collected respectively. Figure 1c presents the representative PL spectra measured at room temperature, where the CW laser has energy of 1.96 eV (details in “Methods” section). Both WSe_2_/Nanocavity and WSe_2_/Sapphire samples exhibit one typical PL emission peak at room temperature. As compared with WSe_2_/Sapphire, the PL intensity from WSe_2_/Nanocavity is significantly enhanced with an average enhancement factor of ~ tenfold, indicative of the plasmonic nanocavity effect. The peak position is blue shifted by 50 meV, attributed to a decrease in binding energy between electrons and holes^25^. As seen from the equation E_PL_ = E_g_-E_b_, where E_PL_ is the exciton emission energy, E_g_ is the electronic band gap and E_b_ is the binding energy between electrons and holes. The plasmon resonance transfers energy to the bonded eletrons and holes (excitons) in WSe_2_, reducing their binding energy, so the emission energy of excitons is enhanced.

Each nanoparticle is spaced well apart from others and these nanoparticles are strongly coupled with the gold substrate individually, enclosing a nanocavity to create a plasmonic system resembling the coupled plasmon dimer. The nanocavity has two main functions: enhancing the localized electric field at the edges of the AuNPs, which influences the carrier dynamics of the in-plane exciton transition dipole of WSe_2_; promoting hot electrons generated in the AuNPs to transfer into the WSe_2_ via z-oriented dipole of the nanocavity; The PL intensity enhancement is attributed to the plasmonic effects brought by the nanocavity, which increases the excitation rate of electrons and holes in the light absorption process and enhances their radiative recombination rate in the emission process via the Purcell effect^2^. The plasmon resonance energy can be determined from the reflectance spectra. Being different from the gold film substrate with no plasmon resonance at visible range (Fig. S1), the nanocavity has strong localized plasmon resonance at 1.68 eV (see Fig. 1d). The key observation here is no energy splitting in PL spectra at room temperature for both WSe_2_/Nanocavity and WSe_2_/Sapphire. Figure 1e presents the simulated spatial distribution of the enhanced electric field, |*E*_*z*_|^2^, inside the monolayer WSe_2_ due to the gap plasmons with electric fields that bridge vertically across the thickness of WSe_2_.

**Figure 2:**
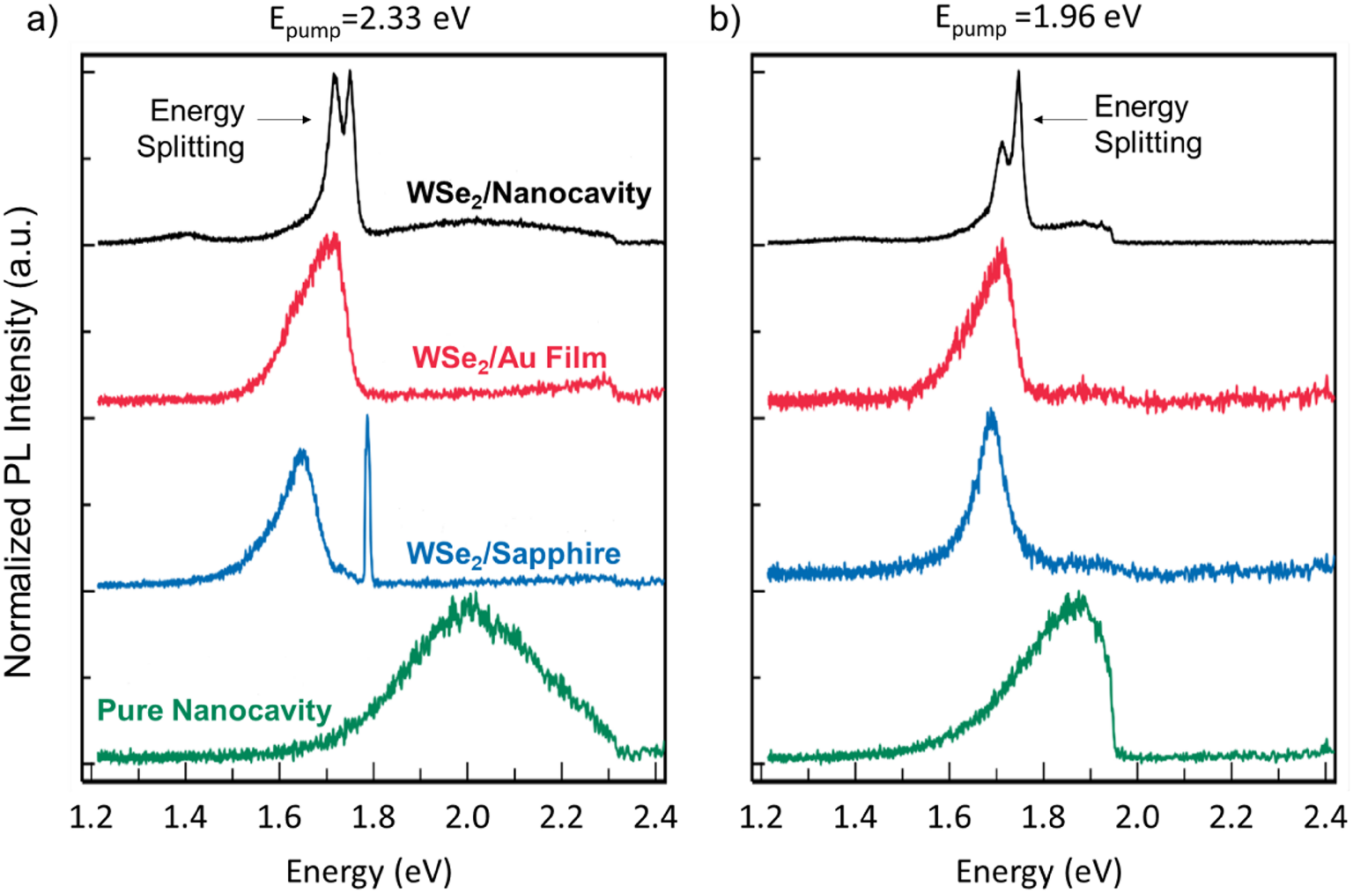
PL emission from monolayer WSe_2_ embedded in different configurations at a temperature of 77 K. The CW pump laser has energy of 2.33 eV (**a**) and 1.96 eV (**b**). The configurations consist of plasmonic nanocavity (WSe_2_/Nanocavity), monolayer WSe_2_ on gold-film substrate (WSe_2_/Au Film), monolayer WSe_2_ on sapphire (WSe_2_/Sapphire) and pure plasmonic nanocavity (Pure Nanocavity). The PL measurements were conducted with a laser power of 20 μW.

A clear energy splitting in the PL spectrum of WSe_2_/Nanocavity is observed at 77 K, as shown in Fig. 2a. The PL spectra from WSe_2_/Nanocavity, WSe_2_ on gold film substrate (WSe_2_/Au Film), WSe_2_/Sapphire and plasmonic nanocavity without WSe_2_ (Nanocavity) were systematically compared by loading the sample in a cryostat cooled by liquid nitrogen to 77 K. Figure 2a presents the PL spectra as being excited by CW laser with energy of 2.33 eV. For WSe_2_/Au Film (red line) and WSe_2_/Sapphire (blue line), there is one typical exciton emission peak at 1.70 eV and 1.65 eV, respectively. No emission due to trions or localized states was observed. The peak at 1.80 eV in WSe_2_/Sapphire is the Raman signal from sapphire. Instead, the WSe_2_/Nanocavity (black line) has well-separated shoulder peaks at 1.72 eV and 1.75 eV, indicating an energy splitting of 33 meV. The peak at 1.7 eV in this hybrid structure is from the plasmon radiative decay of hot electrons in nanocavity, which can be also seen in the pure nanocavity (green line). The observation of energy splitting is further confirmed under laser excitation energy of 1.96 eV and Fig. 2b shows an energy splitting of 35 meV.

**Figure 3:**
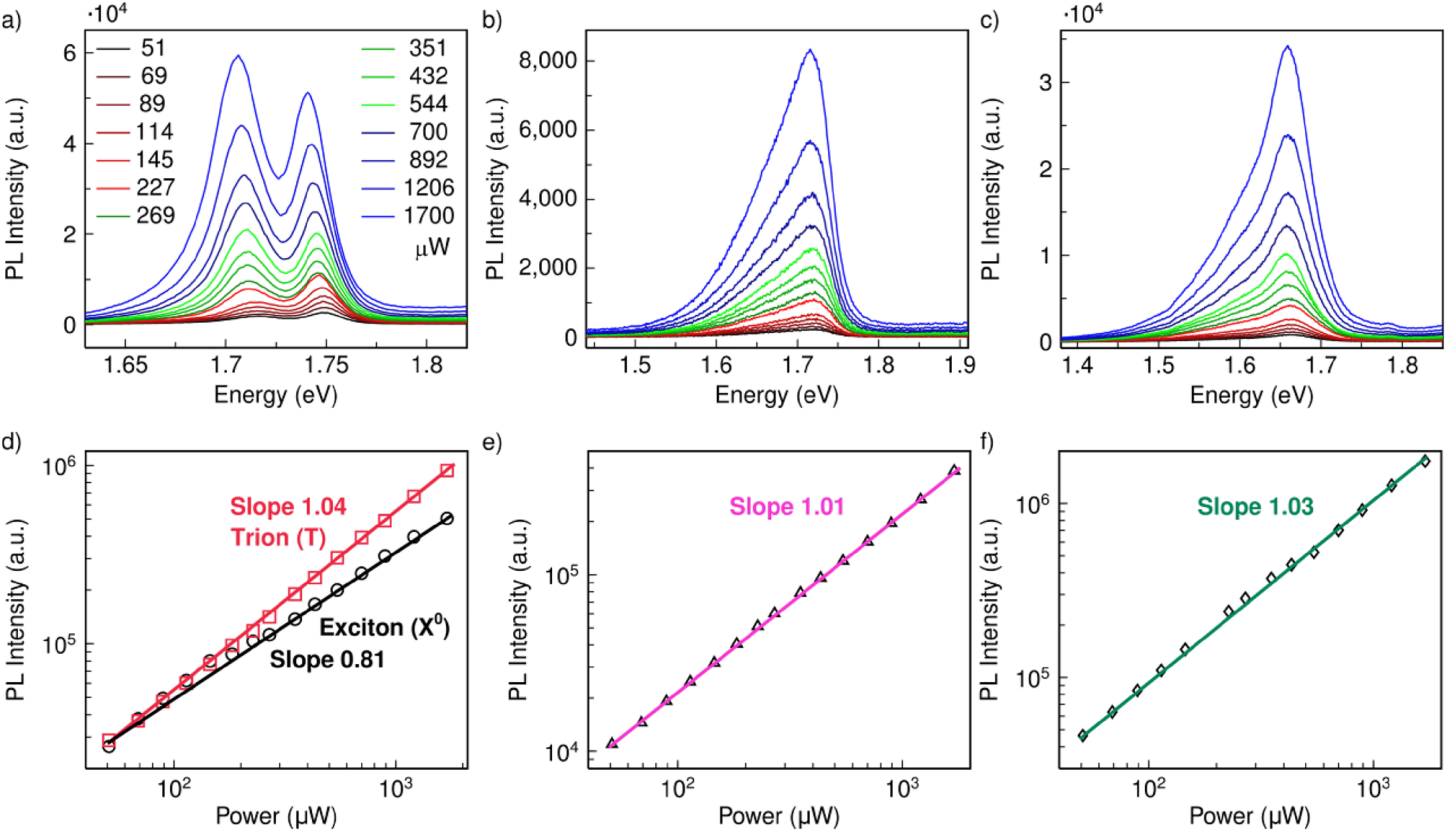
Investigations of the power-dependent PL spectra in different configurations. (**a**) Power-dependent PL spectra of WSe_2_/Nanocavity. (**b**) WSe_2_/Au Film. (**c**) WSe_2_/Sapphire. The excitation laser has energy of 1.96 eV. (**d**–**f**) Integrated PL intensity of WSe_2_/Nanocavity with left peak from 1.65 to 1.73 eV and right peak integrated from 1.73 to 1.76 eV (**d**), WSe_2_/Au Film with peak integrated from 1.55 to 1.77 eV (**e**), and WSe_2_/Sapphire with peak integrated from 1.46 to 1.76 eV (**f**), as a function of laser power.

To determine the mechanism of this splitting energy in the hybrid structure, laser power-dependent PL spectra were measured at 77 K for WSe_2_/Nanocavity, WSe_2_/Au Film and WSe_2_/Sapphire by using laser with energy of 1.96 eV. With increasing laser power from 20 to 1700 μW, the WSe_2_/Nanocavity always exhibited two PL peaks that red-shifted with increasing laser power (Fig. 3a). The right peak occupies the spectra in the low excitation power regime, while the right peak gradually exceeds the left peak and then dominates the spectra with increasing excitation power. The power-dependent variation trend between these two peaks is consistent with previous reports about trion and exciton emission in TMDCs with plasmonic nanostructures^20,26^. In contrast, WSe_2_/Au Film and WSe_2_/Sapphire only exhibited a single broad peak in this wide power range, with no observable shift in peak position (see Fig. 3b and Fig. 3c). This observation suggests that in the WSe_2_/Nanocavity, the strongly localized gap plasmon enables the generation of hot carriers on plasmonic nanostructures, which then transport from the integrated gold plasmonic nanocavity onto WSe_2_, via quantum tunneling through the ligands coating the AuNPs. To be more specific, the Schottky barrier formed between Au and monolayer WSe_2_ with work function of 5.1 eV and 4.3 eV respectively is low (less than 1 eV)^27^, so the hot electron generated by plasmonic excitation of Au NPs can effectively transfer to WSe_2_ via tunneling through the 2-nm thin ligand covering Au NPs. These extra electrons transferred to WSe_2_ leads to the formation of negatively charged excitons (trions) in WSe_2_, and thus trion emission (left peak) can be clearly observed, since the charged trion emission has a lower energy as compared to the charge-neutral exciton emission.

Moreover, the doping effect of plasmonic hot electrons modulates the dielectric permittivity of materials resulting in a continuum redshift of both PL peaks (see Fig. S2). The peak energy of the left peak is ~ 35 meV below the right peak, which is consistent with the trion binding energy of 30–70 meV, as mentioned in previous reports^18,28^. The left peak due to trion has a full-width-at-half-maximum (FWHM) value of ~ 30 meV, which is broader than the right peak (~ 20 meV), according to the fitting of each peak by Lorentz functions (see Fig. S3). Although laser illumination can also cause doping of 2D materials^29–31^, it is not strong enough in our case, since no trion emission or peak shift is obviously observed in WSe_2_/Au Film and WSe_2_/Sapphire. We also exclude the effects of defects and edges in WSe_2_ on the trion emission by comparing the PL spectra of WSe_2_/Nanocavity with the reference sample WSe_2_/Au Film. Although these two samples have comparable defects and edges as they were made from the same piece of WSe_2_/Sapphire and transferred onto the same piece of gold substrate, the trion emission is only observed in WSe_2_/Nanocavity. It proves that the trion emission is caused by the AuNPs instead of defects or edges in WSe_2_. Strain has been reported to influence the electronic structure of 2D materials^32,33^, but there is negligible strain in WSe_2_ with nanocavity as compared with WSe_2_ on gold film substrate, which can be verified by their unshifted Raman peaks (Fig. S4).

The dependence of integrated PL intensity on laser power is shown in Fig. 3d–f. The solid lines are the fitting curves to the data based on a power law:

1 $$ {\text{I}} \propto {\text{P}}^{\alpha } , $$

where I is the integrated PL intensity for a given excitation power P. The extracted exponent factor, α, for WSe_2_/Nanocavity was 1.04 in left peak for trion emission^20^. The exponent factor α is 0.81 for the right peak due to the neutral charged exciton^20^.

In comparison, the exponent factor α for WSe_2_/Au Film and WSe_2_/Sapphire were 1.01 and 1.03 respectively, revealing insights into different dominating recombination processes for each peak. For instance, the peak in WSe_2_/Au Film and WSe_2_/Sapphire are due to excitons respectively, where their photon emission rate was observed to be linearly dependent on the excitation power (I ∝ P). This linear dependence of emission with power in log–log scale is expected for exciton emission as it follows the first-order rate equation for the radiative recombination process. This conclusion is applicable as the WSe_2_ is on gold film substrate or sapphire. However, when WSe_2_ is combined with nanocavity, the behavior of excitons with power is influenced. With increasing power, the excitons partially turn into trions or experience exciton-exciton annihilation, so the emission is suppressed exhibiting a sublinear relation. Specifically, for the right peak of WSe_2_/Nanocavity, the linear dependence at low laser power (≤ 145 μW) indicates its origin from exciton emission. This slope (black line in Fig. 3d) between 351 and 1700 μW then becomes sublinear to infer that the exciton-trion conversion and the many-body effect, exciton-exciton annihilation, begin to play a role.

**Figure 4:**
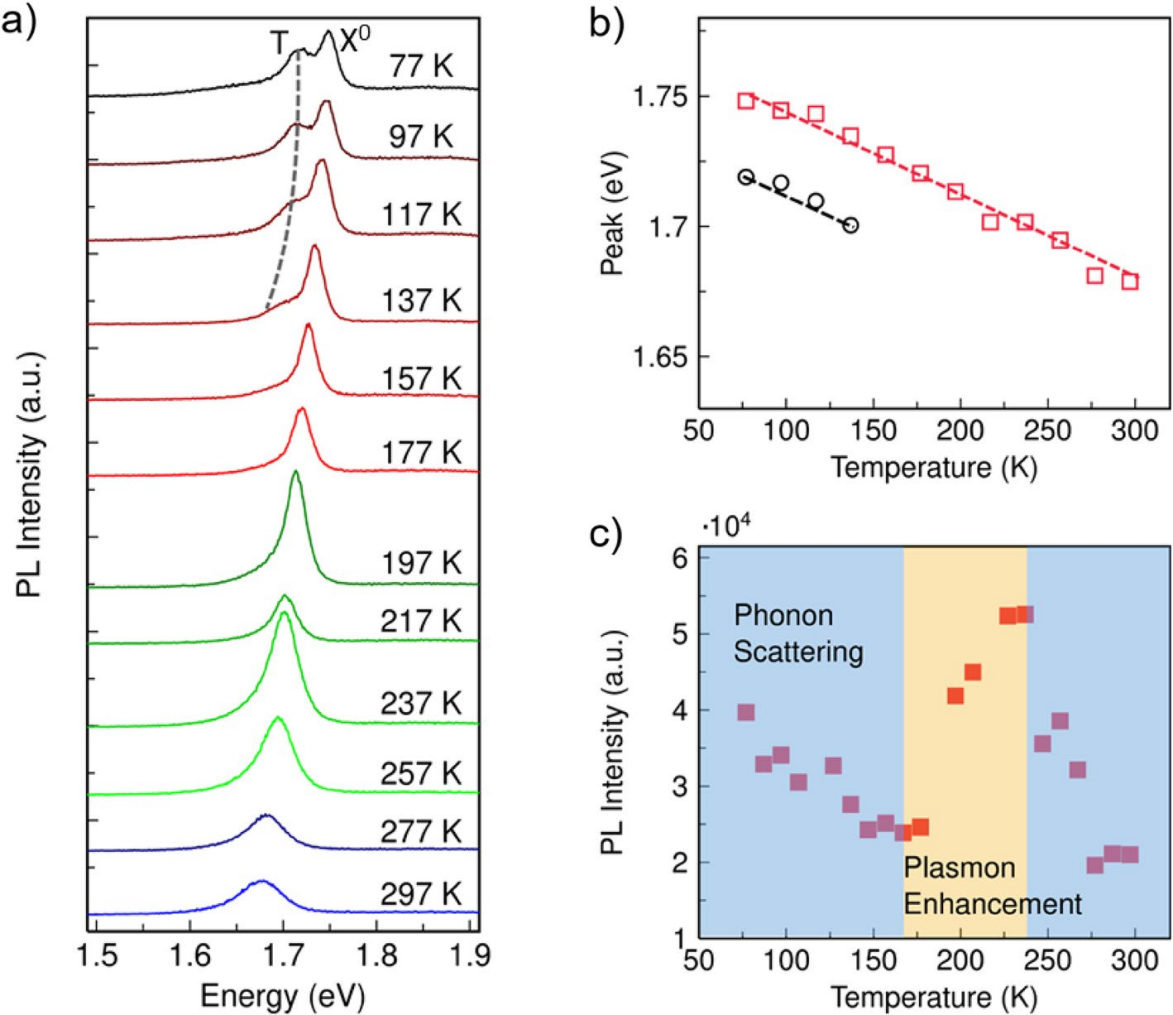
Temperature-dependent PL characteristics. (**a**) Temperature-dependent PL emission from exciton (X^0^) and trion (T) of WSe_2_/Nanocavity. (**b**) PL peak position as a function of temperature. Black dots denote the trion peak and red dots denote the exciton peak, which are fitted by dashed lines. The PL was excited by using laser with energy of 1.96 eV and power of 20 μW. (**c**) Integrated PL intensity of WSe_2_/Nanocavity with peak integrated from 1.62 to 1.78 eV, as a function of temperature. The decreasing and increasing regimes are denoted in blue and orange, respectively. The intensity evolution revealed a combined effect of phonon scattering and plasmon enhancement.

To further verify the origin of the left peak, PL spectra of WSe_2_/Nanocavity between the temperature of 77 and 297 K were carried out by using a laser with energy of 1.96 eV (see Fig. 4a). Neutral exciton (X^0^) and trion (T) emission can be spectrally separated at the temperature of 77 to 137 K. Their energy separation (~ 30 meV) agrees well with reported values^5,17^. As the temperature further increased beyond 137 K, the neutral exciton peak dominated the PL spectrum with a long low-energy tail, which is a signature of the existence of trion emission, and then the tail disappeared. The main exciton emission peak remains at room temperature because of an extremely large exciton binding energy of monolayer WSe_2_ compared with conventional semiconductors. This means that electrons and holes are tightly bound together and they can hardly escape due to thermal fluctuations. Conversely, the trions have smaller binding energies, which can be thermalized much more easily with increasing temperatures. As shown by temperature-dependent peak energy in Fig. 4b, both peaks red-shifted as the temperature increased, and followed each other closely, representing a decreased bandgap. It is noted that the trion emission in our study is only observable at low temperatures < 157 K, but disappears at higher temperatures or room temperature. This is probably because that the thermal effect at high temperatures causes huge loss of the plasmonic hot electrons diffusion and the doped density to WSe_2_ falls below the threshold for the trion emission. Using metallic structure to observe trion emission has also been reported in other 2D TMDCs, e.g., WS_2_^20^, where the plasmon nanostructure resonating with the exciton transition of WS_2_, favors the electron and energy transfer from the plasmon nanostructure to WS_2_, so strong trion emission featured in splitting exciton PL emission can be observed at room temperature and its relative intensity to exciton emission can be tuned by laser power. It is expected that the trion emission in WSe_2_ at room temperature can also be achieved by increasing the doping intensity of the hot electrons from the plasmonic nanostructures via optimizing the design of the nanostructures to align resonance with the exciton transition and increasing the laser power. Figure 4c shows the integrated PL intensity of WSe_2_/Nanocavity as a function of temperature. With increasing temperature, the integrated PL intensity from 1.62 to 1.78 eV exhibits two decreasing regimes (blue) and one increasing regime (orange). Generally, the PL intensity of WSe_2_ decreases with increasing temperature due to the increasing electron–phonon scattering. However, the addition of nanocavity into WSe_2_ breaks the trend as the plasmon resonance is normally stronger at higher temperature. The PL intensity of WSe_2_/Nanocavity makes a trade-off between phonon scattering and plasmon enhancement at 237 K, reaching a maximum value. Therefore, the intensity evolution revealed a combined effect of electron–phonon interactions and a plasmonic effect.

## Conclusions

In summary, we investigated the formation of trions from monolayer WSe_2_, which have been placed inside a plasmonic cavity as formed by gold nanoparticles and a gold-film substrate. A *z*-polarized localized gap plasmon was excited inside the nanogaps between AuNPs and Au film, where this strong localized gap plasmon enables the hot carrier transportation from the integrated gold plasmonic nanocavity onto WSe_2_, resulting in the formation of negatively charged trions. This work is expected to provide a candidate route to manipulate the excitons, which is the essential for the construction of all-exciton information processing circuits and even trion emission directionality control via hybrid plasmon-Mie cavities^34,35^.

## Experimental details and methods

### Growth of monolayer WSe_2_ film

The monolayer WSe_2_ film was grown on sapphire by the chemical vapor deposition (CVD) method^2^. WO_3_ powders of 0.3 g were placed in a ceramic boat located in the center of the furnace and the Se powders were placed in a separate ceramic boat at the upper stream side maintained at 270 °C during the reaction. The sapphire substrates were put at the downstream side. An Ar/H_2_ flowing gas (Ar = 80 sccm, H_2_ = 20 sccm, chamber pressure = 1 Torr) was used to bring the Se and WO_3_ vapors to the targeting sapphire substrates. The center heating zone was heated to 925 °C at a ramping rate of 25 °C min^−1^. When the center heating zone reaches 925 °C, the temperature of the sapphire substrates is 750–850 °C. After reaching 925 °C, the heating zone was kept for 20 min and the furnace was then naturally cooled down to room temperature.

### Transferring of monolayer WSe_2_ film

Then, the WSe_2_ film was transferred to the gold substrate by the wet transfer method^2^. First, the WSe_2_ on sapphire substrate was first coated with a layer of PMMA (950 K, A4) by spin-coating (step 1: 500 rpm for 10 s; step 2: 4000 rpm for 60 s), and then annealed at 130 °C for 2 min. The PMMA at the edges of the sapphire substrate was stroke off with a sharp blade to facilitate the following exfoliation. Then, a NaOH (3 mol L^-1^) solution at 100 °C was used to exfoliate the PMMA-capped WSe_2_ from the sapphire substrate. To remove the etchant and residues, the PMMA-supported WSe_2_ film was transferred to deionized (DI) water. A fresh gold substrate was used to ‘fish out’ the PMMA-capped WSe_2_ film, followed by drying naturally in a fume hood for 12 h. The PMMA was finally removed by acetone vapor and cleaned by isopropyl alcohol (IPA). The gold substrate with WSe_2_ film was uniformly cut into two pieces to ensure fair quality of the WSe_2_ film used in WSe_2_/Au Film and WSe_2_/Nanocavity.

### Preparation of nanocavity structure

The gold substrate was prepared by the template-stripping method^22^. First, 150-nm-thick gold film was deposited onto the silicon substrate by e-beam evaporation. Next, several droplets of the optical adhesive (OA) glue were put onto the sample surface using a pipette. A glass slide was then placed on the top of the OA glue and the OA glue was cured using ultra-violet radiation, followed by template-stripping process. The gold substrate was then stripped from the silicon substrate^23^. After transferring WSe_2_ film on a gold substrate, AuNPs were deposited onto the WSe_2_ film via the directed self-assembly method^22^. AuNPs with oleylamine ligands were suspended in hexane solution and the dip coating speed is 0.3 mm per second so that gold nanoparticles were deposited onto the substrate^24,36^.

### Optical and SEM characterizations

The samples were measured using a confocal micro-photoluminescence setup with CW pump lasers with an energy of 2.33 eV or 1.96 eV, which is focused by a 100 × microscope objective lens (NA = 0.65). PL emission was then collected by the same objective and detected by a charge coupled device. For low temperature measurements, the sample was mounted in a cryostat (Oxford Instrument, Microstat HiRes2) cooled by liquid nitrogen. The optical reflectance spectra of the samples were measured by using a CRAIC UV–VIS-NIR micro-spectrophotometer model QDI 2010 (equipped with a 36 × objective lens with NA = 0.50). Moreover, SEM images were measured by Hitachi SU8220.

## Supplementary Information

Supplementary Information.

## Data Availability

The datasets used and/or analysed during the current study are available from the corresponding author on reasonable request.
