# Supplementary material for: Nanocavity-induced trion emission from atomically thin WSe2
Source: Sci Rep. 2022 Sep 23;12:15861. doi: 10.1038/s41598-022-20226-3 (PMC9508186; doi:10.1038/s41598-022-20226-3)
Supplement: Supplementary file 1 — Supplementary Information. [file 41598_2022_20226_MOESM1_ESM.pdf]

Supporting Information for

Nanocavity-Induced Trion Emission from Atomically Thin WSe_2

Zhuo Wang[1,*], Yuanda Liu[2], Dao Chen[1], Zixuan Wang[1], Mohamed Asbahi[2], Soroosh Daqiqeh Rezaei[3,4], Jie Deng[2], Jinghua Teng[2], Andrew T. S. Wee[5], Wenjing Zhang[1], Joel K. W. Yang[2,6,*] and Zhaogang Dong[2,7,*]

[1]International Collaborative Laboratory of 2D Materials for Optoelectronics Science and Technology of Ministry of Education, Institute of Microscale Optoelectronics, Shenzhen University, Shenzhen 518060, China

[2]Institute of Materials Research and Engineering, A*STAR (Agency for Science, Technology and Research), 2 Fusionopolis Way, #08-03 Innovis, 138634, Singapore

[3]Department of Electrical Engineering, The Pennsylvania State University, University Park, PA, 16802, USA

[4]Materials Research Institute, The Pennsylvania State University, University Park, PA 16802, USA

[5]Department of Physics, National University of Singapore, 2 Science Drive 3, 117551, Singapore

[6]Singapore University of Technology and Design, 8 Somapah Road, 487372, Singapore

[7]Department of Materials Science and Engineering, National University of Singapore, 9 Engineering Drive 1, 117575, Singapore

*Correspondence and requests for materials should be addressed to Z.W (email: wzhuo@szu.edu.cn), Z.D. (email: dongz@imre.a-star.edu.sg) and J.K.W.Y. (email: joel_yang@sutd.edu.sg).

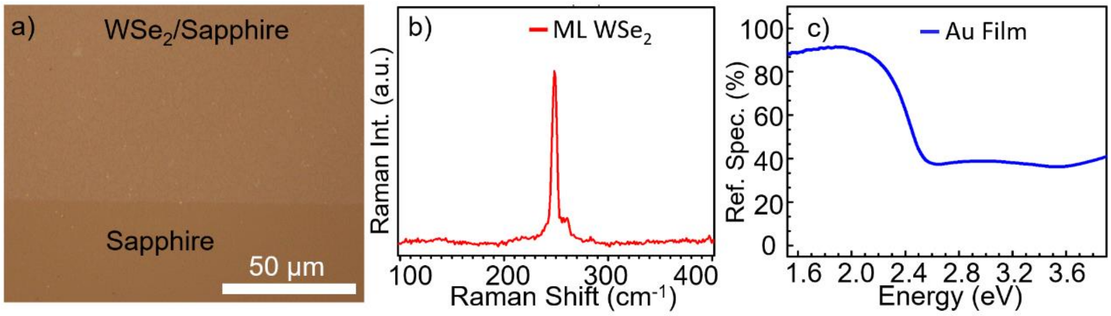

Fig. S1. Characterization of monolayer WSe_2 on sapphire and Au substrate. (a) Optical microscopy (OM) of monolayer WSe_2 grown on sapphire substrate. (b)Raman spectrum of monolayer WSe_2 on sapphire substrate. It has a typical Raman mode at ~ 250 cm^{-1} for monolayer WSe_2. (c) Reflectance spectrum of gold film substrate.

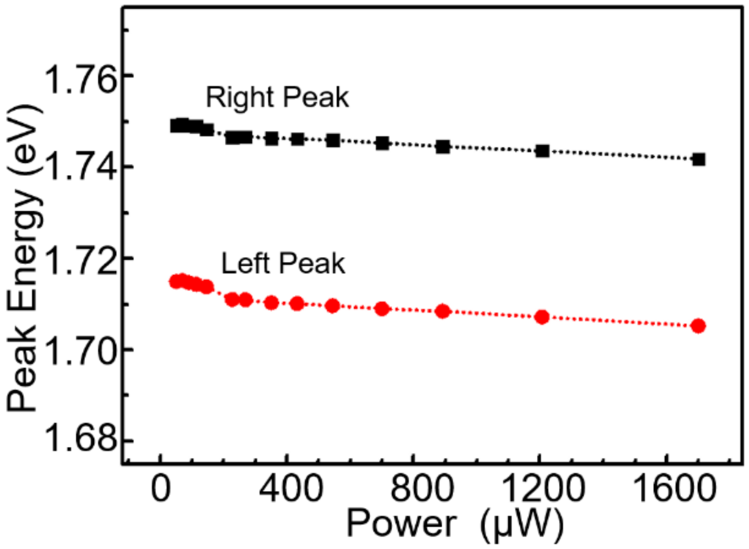

Fig. S2. Laser power dependent peak energy of exciton (right peak) and trion emission (left peak). The splitting energy ranges from 34 to 37 meV.

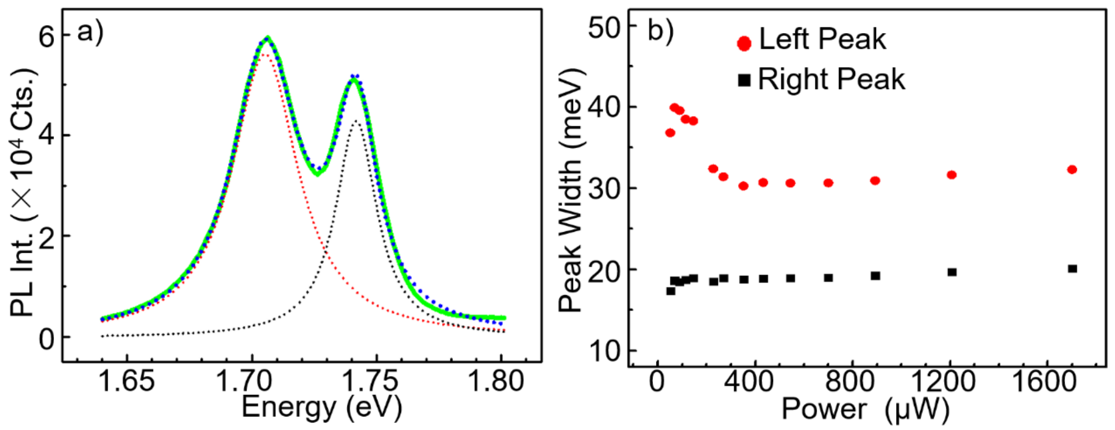

Fig. S3. Lorentz curve fitting of the PL spectra. (a) PL spectrum of WSe_2/Nanocavity excited with laser of power of 1700 μW (green line). It presents an example to show how the two shoulder peaks are fitted by Lorentz function. The left and right peaks are fitted in red and black dots, respectively, and their sum are shown in blue dots. (b) Full-width at half maximum (FWHM) of the left and right PL peaks of WSe_2/Nanocavity at different excitation laser power. Each peak was fitted by Lorentz functions as demonstrated in (a).

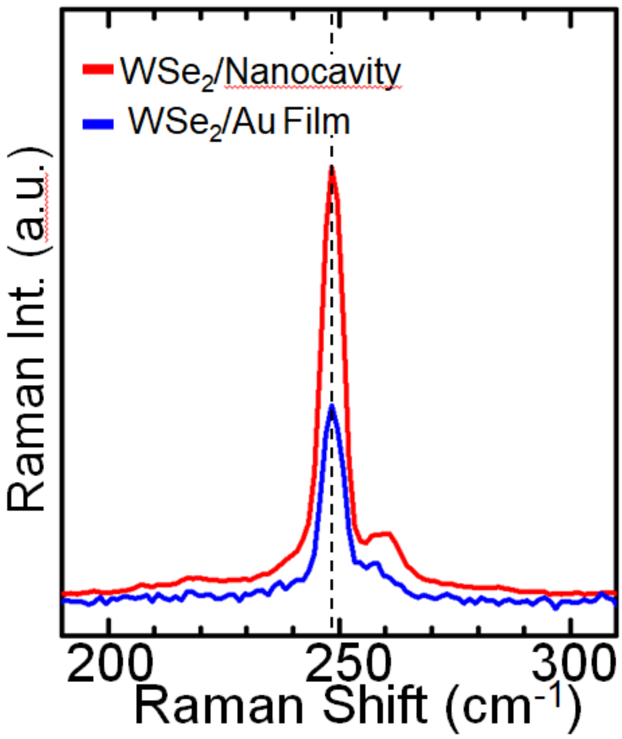

Fig. S4. **Raman spectra of WSe_2/Nanocavity and WSe_2/Au Film excited by 2.33 eV laser.** The black dashed line denotes the Raman peak position of 248.4 cm^{-1}.
